# Update on Vaccine-Derived Polioviruses — Worldwide, January 2014–March 2015

**Published:** 2015-06-19

**Authors:** Ousmane M. Diop, Cara C. Burns, Roland W. Sutter, Steven G. Wassilak, Olen M. Kew

**Affiliations:** 1 Department of Immunization, Vaccines, and Biologicals, World Health Organization, Geneva, Switzerland; 2 Division of Viral Diseases, National Center for Immunization and Respiratory Diseases, CDC; 3 Polio Operations and Research Department, World Health Organization, Geneva, Switzerland; 4 Global Immunization Division, Center for Global Health, CDC

Since the World Health Assembly’s 1988 resolution to eradicate poliomyelitis (1), one of the main tools of the World Health Organization (WHO) Global Polio Eradication Initiative (GPEI) has been the live, attenuated oral poliovirus vaccine (OPV) (2). OPV might require several doses to induce immunity but provides long-term protection against paralytic disease. Through effective use of OPV, GPEI has brought polio to the threshold of eradication. Wild poliovirus type 2 (WPV2) was eliminated in 1999, WPV3 has not been detected since November 2012, and WPV1 circulation appears to be restricted to parts of Pakistan and Afghanistan (1). However, continued use of OPV carries two key risks. The first, vaccine-associated paralytic poliomyelitis (VAPP) has been recognized since the early 1960s (2,3). VAPP is a very rare event that occurs sporadically when an administered dose of OPV reverts to neurovirulence and causes paralysis in the vaccine recipient or a nonimmune contact. VAPP can occur among immunologically normal vaccine recipients and their contacts as well as among persons who have primary immunodeficiencies (PIDs) manifested by defects in antibody production; it is not associated with outbreaks. The second, the emergence of genetically divergent, neurovirulent vaccine-derived polioviruses (VDPVs) was recognized more recently (4). Circulating VDPVs (cVDPVs) resemble WPVs and, in areas with low OPV coverage, can cause polio outbreaks. Immunodeficiency-associated VDPVs (iVDPVs) can replicate and be excreted for years in some persons with PIDs; GPEI maintains a registry of iVDPV cases. Ambiguous VDPVs (aVDPVs) are isolates that cannot be classified definitively (4,5). This report updates previous surveillance summaries (5) and describes VDPVs detected worldwide during January 2014–March 2015. Those include new cVDPV outbreaks in Madagascar and South Sudan, and sharply reduced type 2 cVDPV (cVDPV2) circulation in Nigeria and Pakistan during the latter half of 2014. Eight newly identified persons in six countries were found to excrete iVDPVs, and a patient in the United Kingdom was still excreting iVDPV2 in 2014 after more than 28 years. Ambiguous VDPVs were found among immunocompetent persons and environmental samples in 16 countries. Because the large majority of VDPV case-isolates are type 2, WHO has developed a plan for coordinated worldwide withdrawal of trivalent (types 1, 2, and 3) OPV (tOPV) and replacement with bivalent (types 1 and 3) OPV (bOPV) in April 2016, preceded by introduction of at least 1 dose of injectable inactivated poliovirus vaccine (IPV) into routine immunization schedules worldwide to maintain immunity to type 2 viruses (6).

## Properties of VDPVs

VDPVs are polioviruses whose genetic divergence from the parental OPV strains indicates prolonged replication or circulation (4,5). Poliovirus isolates are grouped into three categories: 1) WPVs; 2) vaccine-related polioviruses (VRPVs); and 3) VDPVs. Current WPVs are genetically unrelated to any vaccine strain. The demarcation between VRPVs and VDPVs is based on the known poliovirus evolution rate. Nucleotide substitutions accumulate in poliovirus genomes at an overall rate of approximately 1% per year and are routinely monitored by sequencing the ~900-nucleotide region encoding VP1, the major poliovirus surface protein. Although nucleotide substitutions might accumulate more rapidly in the early phases of OPV replication, fewer than five VP1 substitutions typically accumulate in the vaccine virus during the normal period of replication in an immunocompetent OPV recipient (4–6 weeks). Based on this rate of nucleotide substitution, type 1 and type 3 isolates that are <1.0% divergent and type 2 isolates that are <0.6% divergent in VP1 sequences from the corresponding vaccine strain are classified as VRPVs. Type 1 and type 3 isolates that are >1.0% divergent or type 2 isolates that are >0.6% divergent in VP1 sequences from the corresponding OPV strain are classified as VDPVs (5). VDPVs are further categorized as 1) cVDPVs when evidence of person-to-person transmission in the community exists; 2) iVDPVs, which are isolated from persons with PIDs; and 3) aVDPVs, which are either clinical isolates from persons with no known immunodeficiency and no evidence of transmission, or sewage isolates that are unrelated to known cVDPVs or iVDPVs and whose source is unknown (5).

## Virologic Testing for VDPVs

All poliovirus isolates are characterized by laboratories of the Global Polio Laboratory Network (5) using a real-time reverse transcription–polymerase chain reaction (rRT-PCR) nucleic acid amplification, targeted to nucleotide substitutions that typically revert to the parental WPV sequence during replication of OPV in the human intestine (7). The rRT-PCR methods are used in 88 of 146 Global Polio Laboratory Network laboratories (5). Candidate VDPVs identified by rRT-PCR screening are sequenced in the VP1 region for definitive analysis; the complete genome is sequenced if required for higher-resolution analysis.

## cVDPVs

**FIGURE 1:**
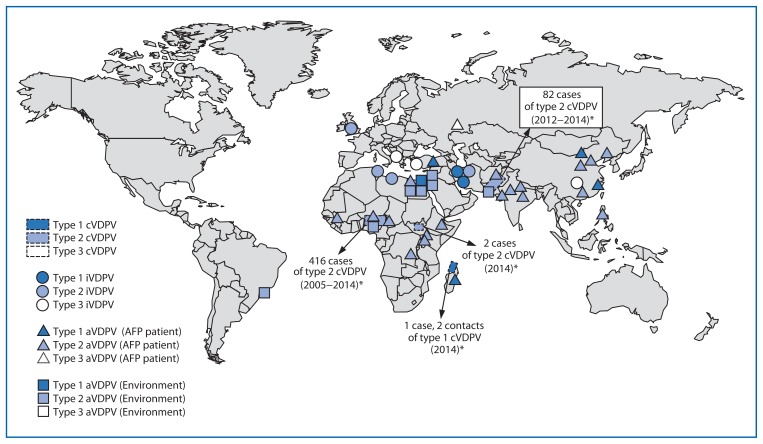
Vaccine-derived polioviruses (VDPVs) detected worldwide, January 2014–March 2015 Abbreviations: cVDPV = circulating VDPV; iVDPV = immunodeficiency-associated VDPV; aVDPV = ambiguous VDPV; AFP = acute flaccid paralysis. * Spread of cVDPVs followed the elimination of the corresponding serotype of indigenous wild poliovirus, but with continued introduction of oral poliovirus vaccine into communities with growing immunity gaps. All of the cVDPV outbreaks were detected first by the laboratory, using sequence data and evolutionary analyses.

**FIGURE 2:**
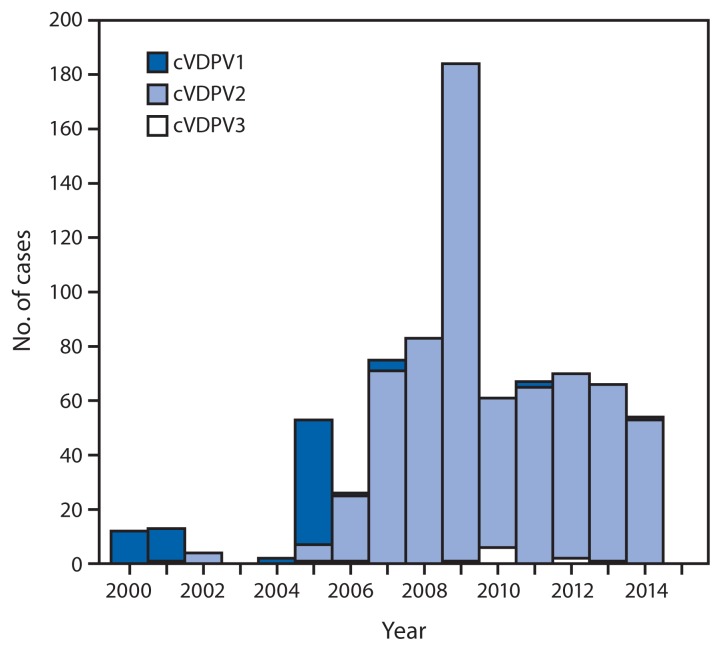
Circulating vaccine-derived poliovirus (cVDPV) cases detected worldwide, by serotype and year, January 2000–March 2015* * Data through March 2015, as available by June 15, 2015.

The number of countries with circulation of indigenously emergent cVDPVs decreased from seven during July 2012–December 2013 (5) to four (Pakistan, Nigeria, Madagascar, and South Sudan) during January 2014–March 2015. Outbreaks associated with indigenous cVDPV2 (Afghanistan, Chad, China, Somalia, and Yemen) and with imported cVDPV2 (Cameroon, Kenya, and Niger) (5) have been interrupted. Although the cVDPV2 outbreak in Pakistan has continued (5), the large outbreak in Nigeria has nearly stopped (5,8), and the two new outbreaks in Madagascar (cVDPV1) and South Sudan (cVDPV2) are small (Table, Figure 1). The most prevalent cVDPVs are type 2 (88.2%), followed by type 1 (10.3%) and type 3 (1.6%). Among the 686 cVDPV cases reported since 2006, >97% were associated with cVDPV2 (Figure 2).

### Madagascar

One cVDPV1 was isolated from an acute flaccid paralysis (AFP) patient in Analalava, Mahjanga Province, on the northwest coast. Circulation is suspected because of the extent of VP1 nucleotide sequence divergence (2.2%) from the parental OPV strain, the absence of immunodeficiency in the AFP patient, and the infection of two nonhousehold contacts with closely related cVDPV1 viruses, as well as the history of repeated cVDPV emergence in Madagascar (5).

### Nigeria

The large indigenous cVDPV2 outbreaks in northern Nigeria, associated with >20 independent cVDPV2 emergences, peaked in 2009 (8), but low-level circulation continued (5). Virus from the major cVDPV2 lineage group that first emerged in 2005 (8) was isolated from 11 AFP patients (most recent onset date: October 14, 2014) and 61 sewage samples (most recent positive sample: March 4, 2015) during the reporting period. Virus from an independent cVDPV2 emergence, apparently originating in Chad in 2012 (5), was isolated from 18 AFP patients (most recent onset date: November 3, 2014) and 32 sewage samples (most recent positive sample: June 18, 2014) in 2014. In addition, four Kaduna State sewage isolates from samples collected from August 2014 through January 2015 had shared nucleotide substitutions at six VP1 positions and the accumulation of VP1 substitutions (0.8%–1.4%) over time (8), both characteristics consistent with cVDPV2s. Circulating VDPV2s were found only in the northern states during the reporting period.

### Pakistan

At least five independent cVDPV2 emergences have occurred in Pakistan since 2012. The emergence associated with most reported cases (71 in Pakistan and four in Afghanistan) was first detected in Killa Abdullah, Balochistan, in August 2012 (5), spread to the insecure North Waziristan Agency in 2013, causing a large outbreak; to parts of Karachi in 2012–2013; and to neighboring Tribal Agencies and Khyber Pakhtunkhwa in 2014. Four cases in Kandahar, Afghanistan, in 2012–2013 were associated with this emergence. The last case from this emergence was reported in June 2014, and the most divergent isolate differed from OPV type 2 at 3.7% of VP1 nucleotide positions. Three additional independent emergences were detected in North Waziristan Agency, associated with five cases in 2013 (0.8%–1.1% VP1 divergence), three during 2013–2014 (0.8%–1.2% VP1 divergence), and two in 2014 (1.1% VP1 divergence), respectively. A fifth cVDPV2 emergence, associated with one AFP case (December 13, 2014), and 29 closely related but nonidentical 2014–2015 sewage isolates (0.8%–2.1% VP1 divergence), has been detected in an insecure part of Karachi, with subsequent introduction into Quetta, Balochistan.

### South Sudan

In September 2014, two cVDPV2 isolates (1.0% VP1 divergence) were identified from patients with AFP in Rubkona, Unity State. The isolates shared three VP1 nucleotide substitutions, consistent with epidemiologic linkage.

## iVDPVs

Since the introduction of OPV in 1961, approximately 100 persons with PIDs worldwide have been found to be excreting iVDPVs, indicating prolonged infection; the majority of these immunodeficiencies were detected only after onset of paralysis. After implementation of intensified surveillance for VDPVs and special studies of iVDPV excretion among persons with PIDs in developing and middle-income countries (9), detection of new iVDPV infections increased from two during January 2008–June 2009, to nine during July 2009–June 2011, and to 12 during April 2011–June 2012, but decreased to 10 during July 2012–December 2013 (5), and to eight during the current reporting period (Table). Like cVDPVs, type 2 iVDPVs are the most prevalent (65%), followed by type 1 (18%) and type 3 (17%). Some patients have heterotypic (i.e., types 1 and 2 or types 2 and 3) iVDPV infections, with the extent of sequence divergence in each isolate of the heterotypic mixture consistent with derivation from a single tOPV source dose (4). Eight new patients with iVDPV infections were reported during January 2014–March 2015 (in addition to the patient with the longest known iVDPV infection, whose infection continued during the reporting period) are described as follows.

### Albania

A boy aged 5 months with X-linked agammaglobulinemia, who first received OPV in March 2014 and developed paralysis in June 2014, his iVDPV3 infection cleared after September 2014.

### China

A boy aged 1 year with PID, who received his third OPV dose in February 2014, his iVDPV3 infection cleared soon after onset of AFP in November 2014.

### Iran

Iran has maintained sensitive clinical and laboratory surveillance to screen persons with PIDs for poliovirus infections. During this reporting period, three patients (two with AFP) were found to be excreting iVDPVs. One was a nonparalyzed child aged 10 months with severe combined immunodeficiency infected with an iVDPV1. Another boy, aged 10 months, with X-linked agammaglobulinemia received OPV in March 2014 and developed paralysis in May 2014; his iVDPV1 infection cleared after August 2014. A boy aged 9 months with PID and infected with iVDPV2 developed paralysis in June 2014 but stopped excreting iVDPVs after September 2014.

### Libya

A nonparalyzed girl aged 1 month with severe combined immunodeficiency traveled to Germany for treatment and was found to be infected with iVDPV2 during November 2013–February 2014; excretion stopped following bone marrow transplantation.

### Tunisia

A nonparalyzed boy aged 11 years with severe combined immunodeficiency was infected with an iVDPV2. He stopped excreting iVDPVs after May 2014.

### Turkey

A nonparalyzed girl aged 1 year with severe combined immunodeficiency was infected with an iVDPV3, which she continued to excrete into December 2014.

### United Kingdom

A man aged 44 years with common variable immunodeficiency was found to be excreting iVDPV2 since 1995. He has no AFP, but the sequence properties of the isolates obtained from serial specimens are consistent with chronic iVDPV2 infection since his last OPV dose at age 7 years.

## aVDPVs

During January 2014–March 2015, aVDPVs were isolated in 16 countries (Table). Detection of aVDPVs in settings with <60% vaccination coverage with 3 doses of polio vaccine might signal cVDPV emergence and potential gaps in surveillance. Some aVDPVs, especially those with limited divergence detected in areas with high vaccination coverage and in patients with no known immunodeficiency, might represent limited spread of OPV or the upper limit of OPV sequence divergence in a single normal vaccine recipient or contact. The most divergent aVDPV was from Brazil, a country with >90% vaccination coverage with 3 doses of polio vaccine. Selected aVDPVs from the reporting period are described as follows.

### Brazil

An aVDPV2 (8.6% VP1 divergence) was isolated from sewage in the Port of São Sebastião, São Paulo, in January 2014. The isolate resembles an iVDPV but is classified as an aVDPV because no immunodeficient source patient has been identified.

### China

Sporadic aVDPVs were isolated in six different provinces during January 2014–March 2015; one aVDPV1 and four aVDPV2s were isolated from AFP patients, and one aVDPV1 was isolated from a healthy child.

### India

Four aVDPV2s (0.7%–1.0% VP1 divergence) were isolated from AFP patients in four different states during January 2014–March 2015.

### Israel

Two isolates of the highly divergent, neurovirulent aVDPV2 first detected in 1998 were detected in sewage samples collected on May 4 and September 22, 2014, and an independent aVDPV2 was found in sewage collected on January 26, 2014.

### Madagascar

An aVDPV1 (3.9% VP1 divergence) was isolated from a patient in Nosy-Varika, Fianarantsoa Province (central east coast), with onset of paralysis on January 31, 2015. Despite a small number of VP1 substitutions shared with the 2014 cVDPV1 isolates from Analalava, this aVDPV1 appears to be an independent emergence.

### Nigeria

Ten aVDPV2s (two from AFP patients, eight from sewage samples, and all with 0.6%–0.7% VP1 divergence) were isolated in the northern states and the Federal Capital Territory during the reporting period.

### Pakistan

Fifteen aVDPV2s isolates (10 from AFP cases/contacts, four from sewage samples, and all with 0.8%–2.3% VP1 divergence) were isolated during January 2014–March 2015. The most recent aVDPV2 isolates were from the Khyber Agency (two AFP cases in February 2015 and 0.8% VP1 divergence), and Peshawar, Khyber Pakhtunkhwa (from a January 2015 sewage sample, with 0.8% VP1 divergence).

#### Discussion

During January 2014–March 2015, the size and geographic distribution of cVDPV outbreaks further declined since July 2012–December 2013. However, new cVDPV2 lineages have emerged in both Nigeria and Pakistan in settings of insecurity and widening immunity gaps to cVDPV2. Inclusion of more tOPV rounds in the steadily improving supplementary immunization activities (SIAs)* and ensuring increased access to unimmunized children are important factors in controlling cVDPV2 outbreaks. The new outbreaks in Madagascar and South Sudan underscore the importance of maintaining high population immunity to all polioviruses and of sensitive AFP surveillance.

Although expanded testing of sewage samples for the presence of poliovirus (environmental surveillance) in Nigeria and Pakistan has increased the sensitivity of poliovirus detection, especially cVDPV2, which has a 10-fold lower case-to-infection ratio than WPV1 (4), it also presents new logistical and technical challenges to the Global Polio Laboratory Network, because VDPVs must be detected within the complex mixtures of polioviruses and other enteric viruses present in sewage. The rRT-PCR screening for VDPVs must be capable of recognizing the small number of genetic differences distinguishing VDPVs from the closely related VRPVs, which are currently of little public health interest, while striking a balance between sensitivity and specificity not required for the identification of WPVs, which are readily distinguishable from VRPVs and VDPVs (7); the requirement for high specificity has resulted in an increased need for nucleotide sequencing.

Interpreting the virologic data presents additional challenges: one VDPV isolate from an AFP patient might signal hundreds to thousands of inapparent cVDPV infections, whereas multiple VDPV sewage isolates might derive from a single iVDPV infection. Environmental cVDPV isolates can be recognized by their close genetic relationship with known cVDPVs from one or more AFP patients or by local detection of closely related VDPVs over several months that show progressive divergence from the parental OPV strain. These latter environmental cVDPVs are distinguishable by their sequence properties from those environmental aVDPVs (4) which very likely signal the presence of a chronic iVDPV excretor in the community. Indeed, highly divergent environmental aVDPVs that are probably iVDPVs from unidentified chronic excretors have been detected in five countries, most recently in Brazil. Detection of less divergent environmental VDPVs (especially VDPV2s) without linkage to known infected persons presents the greatest challenges for epidemiologic interpretation.

**Summary**

What is already known on this topic?

Genetically divergent vaccine-derived polioviruses (VDPVs) are detected by poliovirus surveillance and have biologic properties indistinguishable from wild polioviruses. High polio vaccination coverage can prevent circulating VDPV (cVDPV) outbreaks, but prolonged immunodeficiency-associated VDPV (iVDPV) infections will occur as long as oral poliovirus vaccine (OPV) is used.

What is added by this report?

The intensity of cVDPV transmission fell after mid-2014. Recent cVDPV outbreaks in Afghanistan, Chad, Somalia, and Yemen have apparently stopped, and the large outbreak in Nigeria has nearly stopped. Virus of the major cVDPV emergence in Pakistan was last detected in June 2014, but low-level circulation of a new emergence was detected into 2015. New, possibly small, outbreaks were detected in Madagascar and South Sudan. Nine new prolonged iVDPV infections in seven countries were detected, either by characterization of isolates from patients with acute flaccid paralysis (AFP) or by intensified search for iVDPV excretion among persons with primary B-cell immunodeficiencies. Since 2006, >97% of cVDPVs detected have been type 2.

What are the implications for public health practice?

Circulating VDPV outbreaks can be prevented and controlled by high OPV coverage. By contrast, only cessation of OPV use will prevent prolonged iVDPV infections. WHO has responded to the continued global type 2 VDPV risk by incorporating the following into its new strategic plan: 1) shifting from trivalent OPV to bivalent OPV (types 1 and 3) by April 2016; 2) including ≥1 dose of inactivated poliovirus vaccine into routine immunization schedules worldwide; 3) maintaining strategic stockpiles of monovalent OPV; 4) developing a robust acute flaccid paralysis and poliovirus surveillance and response capacity; and 5) encouraging development of antiviral drugs to clear prolonged iVDPV infections.

Special studies in several countries to search for VDPV infections among patients with PIDs have increased the number of known iVDPV excretors, while documenting the infrequency of iVDPV infections, even among persons with PIDs (9). Global AFP surveillance and environmental surveillance have proven most sensitive in detecting prolonged iVDPV excretion.

In view of the rising incidence of cVDPV2 outbreaks more than a decade after the last known WPV2 case, GPEI has incorporated the coordinated worldwide withdrawal of tOPV and replacement with bOPV into its new strategic plan, with the ultimate goal of stopping all OPV use (6). The switch from tOPV to bOPV, planned for April 2016, is predicated on the absence of any known cVDPV2 transmission (6). The absence of any reported cases associated with cVDPV2 in 2015 (all cVDPV2 isolates through March 2015 were from environment samples) and the current low frequency of cVDPV2 detection worldwide is encouraging. To ensure that VDPV emergence is minimized and that any VDPV infections are detected, it will be essential to continue efforts to strengthen routine immunization services and to strengthen AFP and poliovirus surveillance during 2015. Most countries will incorporate at least 1 dose of IPV into routine childhood immunization schedules in 2015 (6).

Replacement of tOPV with bOPV will greatly reduce the risk for cVDPV2 outbreaks, and global cessation of OPV use will ultimately prevent all cVDPV outbreaks and all new iVDPV infections (6). However, a small number of persons with chronic iVDPV infections, as exemplified by the non-AFP common variable immunodeficiency patient from the United Kingdom, might continue to excrete poliovirus for a decade or more after receipt of the last OPV dose. Therefore, maintenance of high levels of population immunity through comprehensive IPV coverage will be necessary to protect against iVDPV becoming a source of spread in the community. Detection of chronic iVDPV excretors in all countries (9) and development of antivirals to clear chronic iVDPV infections are also important (10).

## Figures and Tables

**TABLE:** Vaccine-derived polioviruses (VDPVs) detected worldwide, January 2014–March 2015

Category	Country	Year(s) detected*	Source of isolates (total cases or specimens)†	Serotype	No. of isolates§			VP1 divergence from Sabin OPV strain (%)	Routine coverage with 3 doses of polio vaccine (%)¶	Estimated duration of VDPV replication**	Current status (date of last outbreak case, last patient isolate, or last environmental sample)

					Cases	Contacts	Non-AFP source				
cVDPV	Madagascar	2014	AFP patient	1	1	2	—	2.2	73	2 yrs	September 29, 2014
	Nigeria	2005–2015	Outbreaks (394 total cases)††	2	11	—	61	0.7–8.4	67	10 yrs	March 4, 2015§§
	Nigeria	2013–2014	Importation¶¶ (22 total cases)	2	18	—	32	1.2–3.9	67	3 yrs	November 3, 2014
	Pakistan	2012–2015	Outbreaks (82 total cases)	2	18	3	26	0.7–3.7	72	3 yrs	March 28, 2015***
	South Sudan	2014	2 cases	2	2	—	—	1.0	50	~1 yr	September 12, 2014
iVDPV	Albania	2014	AFP patient XLA	3	1	—	—	0.7–1.0	99	6 mos	September 12, 2014
	China	2014	AFP patient	3	1	—	—	1.4	99	~1 yr	November 26, 2014
	Iran	2014	Non-AFP SCID	1	—	—	1	2.4	98	10 mos	April 15, 2014
		2014	AFP patient XLA	1	1	—	—	1.8		1.5 yrs	August 2, 2014
		2014	AFP patient PID	2	1	—	—	0.7		<1 yr	September 13, 2014
	Libya†††	2014	Non-AFP SCID	2	—	—	1	0.7–1.0	95	4 mos	February 7, 2014
	Tunisia	2014	Non-AFP SCID	2	—	—	1	1.0	98	~1 yr	May 2014
	Turkey	2014	Non-AFP SCID	3	—	—	1	1.2	98	1.4 yrs	February 17, 2015
	UK	2014	Non-AFP CVID	2	—	—	1	17.9	96	>28 yrs	June 22, 2014
aVDPV	Brazil	2014	Environment	2	—	—	1	8.6	99	8 yrs	January 2014
	Chad	2015	AFP patient	2	1	—	—	0.8	50	<1 yr	January 8, 2015
	China	2014–2015	AFP patient	1	1	—	—	1.1	99	~1 yr	March 20, 2015
		2014–2015	AFP patients	2	4	—	—	0.7–2.4		<1 yr; 2 yrs	March 21, 2015
		2014	Non-AFP patient	1	—	—	1	1.1		~1 yr	October 2014
	DRC	2014	AFP patient	2	1	—	—	1.1	70	1 yr	January 15, 2015
	Egypt	2014	AFP patient	2	1	—	—	1.0	97	~1 yr	April 19, 2014
		2014	Environment	1	—	—	2	1.1; 2.7		1 yr; 2.5 yrs	April 20, 2014
		2014–2015	Environment	2	—	—	2	0.7		<1 yr	February 4, 2015
	Ethiopia	2014–2015	AFP patient	2	1	—	—	0.7–0.9	70	<1 yr	March 5, 2015
	Guinea	2014	AFP patient	2	1	—	—	1.3	64	~1 yr	August 30, 2014
	India	2014–2015	AFP patients	2	4	—	—	0.7–1.0	70	~1 yr	February 26, 2015
	Israel	1998–2014	Environment	2	—	—	2	>15%	94§§§	>15 yrs	September 22, 2014
		2014	Environment	2	—	—	1	0.7		<1 yr	January 26, 2014
	Madagascar	2015	AFP patient	1	1	—	—	3.9	73	2 yrs	January 31, 2015
	Nigeria	2014	AFP patients	2	2	—	—	0.7	67	<1 yr	April 5, 2014
		2014–2015	Environment	2	—	—	8	0.7–1.4		≤1 yr	March 9, 2015
	Pakistan	2014–2015	AFP patients	2	9	1	—	0.8–2.3	72	≤1 yr; 2 yrs	February 9, 2015
		2014–2015	Environment	2	—		6	0.8–1.4		≤1 yr	January 2015
	Philippines	2015	AFP patient	2	1	—	—	0.8	88	<1 yr	December 18, 2014
	Russia	2014	AFP patient	3	1	3	—	1.1	98	~1 yr	July 10, 2014
	Turkey	2014	AFP contact	1	—	1	—	1.0	98	~1 yr	May 8, 2014
	Uganda	2014	AFP patients	2	2	—	—	0.7	82	<1 yr	August 13, 2014

**Abbreviations:** cVDPV = circulating VDPV; iVDPV = immunodeficiency-associated VDPV; aVDPV = ambiguous VDPV; OPV = oral poliovirus vaccine; IPV = inactivated poliovirus vaccine; AFP = acute flaccid paralysis; PID = primary immunodeficiency; SCID = severe combined immunodeficiency; XLA = X-linked agammaglobulinemia; CVID = common variable immunodeficiency; DRC = Democratic Republic of the Congo.

* Total years detected and cumulative totals for previously reported cVDPV outbreaks (Nigeria and Pakistan).

† Outbreaks list total cases clearly associated with cVDPVs. Some VDPV case isolates from outbreak periods might be listed as aVDPVs.

§ Total cases for VDPV-positive specimens from AFP cases and total VDPV-positive samples for environmental (sewage) samples.

¶ Based on 2013 data from the World Health Organization (WHO) Vaccine Preventable Diseases Monitoring System (2014 global summary) and WHO–United Nations Children’s Fund (UNICEF) coverage estimates, available at http://www.who.int/immunization/monitoring_surveillance. National data might not reflect weaknesses at subnational levels.

** Duration of cVDPV circulation was estimated from extent of VP1 nucleotide divergence from the corresponding Sabin OPV strain; duration of immunodeficiency-associated VDPV replication was estimated from clinical record by assuming that exposure was from initial receipt of OPV; duration of ambiguous VDPV replication was estimated from sequence data.

†† Count does not include 29 cases with <10 substitutions in VP1 detected before 2010.

§§ The most recent isolate was from an environmental sample.

¶¶ Importation from Chad.

*** The most recent isolate was from an environmental sample.

††† The VDPV was detected and characterized in Germany where the patient had gone for treatment.

§§§ Value for routine IPV immunization in 2013. Israel conducted two rounds with bivalent OPV in response to detection of imported wild poliovirus type 1 from environmental samples.

## Footnotes

* SIAs are mass vaccination campaigns conducted in a short period (days to weeks) during which a dose of OPV is administered to all children aged <5 years, regardless of previous vaccination history. Campaigns can be conducted nationally or in portions of a country.
